# Development and Validation of a System for the Assessment and Recovery of Grip Force Control

**DOI:** 10.3390/bioengineering10010063

**Published:** 2023-01-04

**Authors:** Martina Lapresa, Clemente Lauretti, Francesco Scotto di Luzio, Federica Bressi, Fabio Santacaterina, Marco Bravi, Eugenio Guglielmelli, Loredana Zollo, Francesca Cordella

**Affiliations:** 1 Research Unit of Advanced Robotics and Human-Centred Technologies, Department of Engineering, Università Campus Bio-Medico di Roma, Via Álvaro del Portillo, 21, 00128 Roma, Italy; 2 Unit of Physical Medicine and Rehabilitation, Università Campus Bio-Medico di Roma, Via Álvaro del Portillo, 21, 00128 Roma, Italy

**Keywords:** grip force control, exoskeleton, virtual reality, gamification, tracking task, biofeedback, hand rehabilitation and assessment

## Abstract

The ability to finely control hand grip forces can be compromised by neuromuscular or musculoskeletal disorders. Therefore, it is recommended to include the training and assessment of grip force control in rehabilitation therapy. The benefits of robot-mediated therapy have been widely reported in the literature, and its combination with virtual reality and biofeedback can improve rehabilitation outcomes. However, the existing systems for hand rehabilitation do not allow both monitoring/training forces exerted by single fingers and providing biofeedback. This paper describes the development of a system for the assessment and recovery of grip force control. An exoskeleton for hand rehabilitation was instrumented to sense grip forces at the fingertips, and two operation modalities are proposed: (i) an active-assisted training to assist the user in reaching target force values and (ii) virtual reality games, in the form of tracking tasks, to train and assess the user’s grip force control. For the active-assisted modality, the control of the exoskeleton motors allowed generating additional grip force at the fingertips, confirming the feasibility of this modality. The developed virtual reality games were positively accepted by the volunteers and allowed evaluating the performance of healthy and pathological users.

## 1. Introduction

The occurrence of neuromuscular or musculoskeletal disorders can seriously affect the ability to finely control and modulate hand grip forces [1,2], and a prompt rehabilitative treatment is needed to recover the degree of autonomy requested to perform the activities of daily living (ADLs) [3]. In traditional rehabilitative therapy, the therapists actively move the patient’s limbs and the patient attempts to initiate movements autonomously. Many studies demonstrate that quantity, duration, intensity, and content (e.g., task-oriented exercises) of training sessions considerably influence the mechanism of neuroplasticity and the relearning of motor skills. However, with this traditional approach, training sessions are often limited in time, and the potential of a prompt rehabilitative treatment is not fully exploited [4,5]. Nowadays, technological solutions can be adopted for administering robot-aided rehabilitation in order to enhance the benefits of traditional therapy [4,5,6,7,8]. Indeed, a robot can move and guide the patient’s hand in executing task-oriented and repetitive motor exercises, and quantitatively tune the intensity of the therapy according to the patient’s condition. Regarding the functional assessment of the patient, a robotic device allows quantitatively and objectively evaluating the observed parameters and reducing human-related errors [6]. Normative parameter values could be subject-specific and measured on the contralateral healthy limb (e.g., in case of hemiparesis, and typically done in mirror therapy) or relative to the healthy population (normative RoM for a particular joint, normative movement speed, normative forces, etc.).

In addition, the use of a robotic system combined with virtual reality (VR) can improve the rehabilitation outcome [4,7,8]. In fact, the use of VR and the gamification of the therapy (i.e., the incorporation of game principles into the therapy so as to encourage participation) allows actively engaging with and motivating the patient during the therapy through various types of feedback, thereby improving therapy outcomes [8,9]. The biofeedback is an essential factor to be considered for enhancing motor learning, and it can be provided through the use of VR. It is a technique which allows perceiving voluntary or involuntary processes occurring in the body to manipulate them through conscious mental control. Therefore, providing the patient with biofeedback, either during or after the therapy, can improve the outcome of the treatment and promote neuroplasticity [1,4].

The articular range of motion (RoM) and the force exerted by the hand when grasping and manipulating objects are two of the most significant parameters in the evaluation of hand functionality [10,11,12,13]. In particular, the ability to finely control and scale the grip forces can be greatly compromised in patients with neuromuscular or musculoskeletal disorders [1]. Therefore, an objective and accurate measurement of the sub-maximal grip forces exerted in grasping tasks is essential to assessing the improvements in the patient and to evaluate the effects of the treatment [1]. Moreover, the objective of the rehabilitative therapy is the recovery of the capability to perform ADLs, so it is paramount to train the patient to perform different types of grasps, promote the interaction with real objects in task-oriented exercises, and to train and assess the ability of the subject to exert and modulate sub-maximal grip forces, which are exploited to manipulate objects in ADLs [11].

The training and assessment of grip force control (i.e., the ability to finely control and modulate the exerted grip forces) can be efficiently performed with tracking tasks. In a tracking task, the patient applies a force onto a sensorized object, a handle or a robotic device, and information about the intensity of this force is routed back by means of a simple desktop environment or a more complex virtual environment. A target trajectory must be tracked as accurately as possible by controlling the exerted grip force [14]. Previous works proposed tracking tasks for the evaluation and training of grip force control. In [1], the authors described and tested a grip force training system that enabled them to improve grip force control in 8 out of 10 post-stroke patients. In the proposed task, a target blue ring moved vertically in the screen to generate, as time passed, a reference trajectory to be tracked. When the force was applied onto a grip force measuring device, a red dot controlled by the patient’s force input moved upwards. Similarly, when the force was released, the red dot moved downwards. In this way, the patient could train his/her force modulation capability trying to reach and track the blue ring with the red dot. In [15], the authors pointed out that the power grip force tracking tasks developed to assess grip force modulation proved to be feasible for quantifying grip force control accuracy in mildly to severely affected hemiparetic stroke patients.

To the best of our knowledge, many devices allow evaluating finger RoM in a very accurate way [16,17,18,19], but only a small number of them provide measurement of the force exerted by the fingers during the grasping tasks, even though both parameters are paramount to assessing hand functionality. Moreover, the existing devices mostly consist of end-effector systems that exploit instrumented objects to measure the force exerted by the hand. For example, AMADEO^®^ and PABLO System^®^ (Tyromotion GmbH, Graz, Austria) can be mentioned. AMADEO^®^ supports the flexion/extension of each finger and provides measures of the finger’s RoM and force, and it was used in the literature to administer robotic-assisted therapy based on VR games [20]. PABLO System^®^ allows performing grasping movements and evaluating the force exerted by the whole hand by means of an instrumented object. It can be used with VR games to engage the patient during therapy. Other end-effector devices, such as the HapticKnob [21] and the HandCARE [22] were exploited in research to administer VR-based hand force rehabilitation therapy. However, the shape of the end-effector, the instrumented object, can constrain the types of grasp that can be executed: for example, a cylindrical end-effector with a given diameter only allows training power grasps and does not allow the possibility of training precision grasps. Moreover, instrumented objects (e.g., electrical dynamometers) typically do not allow determining the magnitude of the force exerted by each finger [11].

Exoskeletal devices, instead, do not constrain the palm of the hand and allow monitoring each finger during interactions with real objects of different shapes and dimensions and to train various types of grasps. Providing exoskeletons with the possibility of sensing forces exerted by each finger could allow training grip force control and modulation capability in grasping tasks of a variety of objects to simulate ADLs, and enable specific evaluation and intervention on the impairment [3]. In the literature, some examples of exoskeletal devices able to measure grip forces exerted by each finger can be found. The hand exoskeleton developed in [23] for assistive and rehabilitative purposes can identify and prevent object slippage and adapt grip geometries to the object’s properties thanks to the information coming from force sensors at the fingertips. The device developed in [24] monitors the user’s finger movement efforts thanks to force sensors and allows augmenting the wearer’s force production. However, despite the importance of grip force assessment and training, the above systems do not exploit the grip force measurement to train the patient’s grip force control and do not take advantage of any kind of biofeedback to engage the patient during the rehabilitative treatment.

This paper presents a system for the assessment and recovery of grip force control that can be fully integrated into a commercial exoskeleton for hand rehabilitation. The main goals of this work were: (i) To develop and perform preliminary tests of an active-assisted modality on healthy subjects as a proof-of-concept. This modality is to be further developed and tested on the clinical population, for administering an active-assisted training in which the patient can be assisted by the machine to reach target force values. (ii) To develop and test with healthy and pathological subjects a VR game based on tracking tasks for the recovery and assessment of grip force control. Both goals used force information recorded from force sensors embedded at the fingertips of the exoskeleton.

The proposed system has been developed and implemented on the Gloreha Sinfonia glove (Idrogenet, Italy) [3] for demonstration purposes, but it could be adapted to other similar exoskeletal machines, thereby enabling grip strength training in addition to motion training. Moreover, the proposed solution is suitable for both therapy and assessment purposes, and two different modes of operation are selectable: (i) active-assisted training to assist the user in reaching target grip force values and (ii) biofeedback with VR games for the assessment and recovery of grip force control.

The paper is organized as follows: Section 2 describes the materials and methods, i.e., the instrumentation of the exoskeletal device, the active-assisted modality and the VR game modality, the experimental protocol and setup used to test the modalities, the considered performance indicators, and the statistical analysis. Section 3 reports the results of the experimental validation, which are discussed in depth in Section 4. Conclusions and future work are provided in Section 5.

## 2. Materials and Methods

In this work, an exoskeleton for hand rehabilitation was instrumented, and two different modes of operation were developed for the assessment and training of users’ grip force control. In the modality “active-assisted training”, the actuators of the device are exploited to generate additional grasp force during the force training and assist the user in reaching target force values. In the modality “VR game for training and evaluation”, instead, the exoskeleton with embedded force sensors is used to record the forces exerted by each finger during the execution of exercises purposely developed to improve and assess the force modulation capability. For each modality, custom-made software solutions and graphical user interfaces (GUIs) were developed to control the device and provide biofeedback.

### 2.1. The Instrumented Hand Exoskeleton

**Figure 1:**
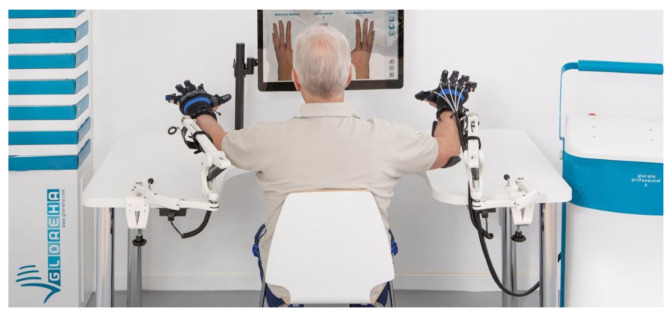
Gloreha Sinfonia (www.gloreha.com/sinfonia (accessed on 18 June 2020)).

**Figure 2:**
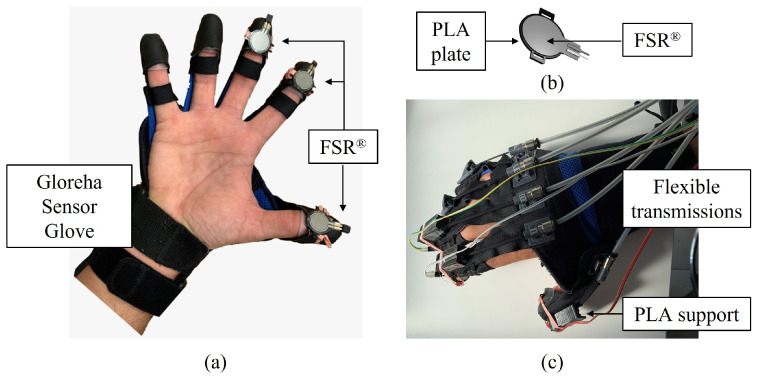
(**a**) Gloreha sensor glove with force-sensing resistors (FSR^®^) embedded in the fingertips of the first, second, and third finger. (**b**) CAD detail of the sensor interface: the 3D printed PLA plate allows uniformly distributing the force on the sensitive area of the sensor. (**c**) Instrumented glove with flexible transmissions mounted on the back, which transmit forces and displacements to the fingertips.

The exoskeletal device used to develop the force-training experimental system is the Gloreha Sinfonia (Idrogenet, Italy), which is shown in Figure 1 [25]. It is typically used to deliver rehabilitative treatment for the recovery of functional hand RoM, which is measured by means of resistive flex sensors embedded in the glove. The system can also actively assist the patient in the execution of flexion/extension movements of the fingers according to the RoM information, thanks to the five supplied linear, permanent magnetic DC motors with flexible transmissions, which transmit forces, displacements, and velocities to the fingertips. Transmissions are mounted on the back of the exoskeletal glove and can be inserted into cloth sleeves that extend from the dorsum of the hand to the fingertips (Figure 2c). Gloreha Sinfonia motors can be controlled positionally by setting the incremental movement step as percentage of the motor’s maximum, from 0% (extended hand) to 100% (flexed hand). The Gloreha Sinfonia glove does not cover the palm, as shown in Figure 2a. Leaving the palm free to have contact with objects is less constraining for hand movements and lets the glove holder perceiving sensory feedback. Furthermore, it is useful to avoid any grasping reflex, which by inducing a force closure of the hand, could favor the hyper tonus insurgence [3]. The visual feedback consisting of interactive games allows engaging the patient during the therapy (Figure 1).

However, this system includes neither the monitoring of the grasping force, nor hand force training. Therefore, we aimed at widening Gloreha Sinfonia’s functions to include force training and force assessment by embedding force sensors in the glove fingertips.

The following technical specifications were considered for the force sensors:

- Thickness: less than $5\times 10^{-4}$ m to guarantee flexibility and not to affect acceptability and usage comfort [26];
- Dimensions: maximum $2\times 10^{-2}$ m diameter;
- Force range (comparable to the one detected in hand rehabilitation training): 0–25 N [10].

Force-sensing resistors satisfied the technical specifications. In particular, the piezoresistive sensor FSR^®^ Model 402 Short Tail (Interlink Electronics, Camarillo, CA USA) was selected. It has a thickness of $4\times 10^{-4}$ m, a diameter of $1.83\times 10^{-2}$ m, a force sensitivity range of 0.2–20 N, and continuous force resolution. Moreover, this type of sensor is low cost, requires very simple conditioning electronics [26], and has a good shock resistance, thereby being suitable for wearable applications.

Three FSR^®^ were embedded in the fingertips of the thumb, index finger, and middle finger of the exoskeletal glove, in order to limit the complexity of the prototype. As also highlighted in research studies that investigated force synergies [27,28], they are the fingers mostly involved in daily grasping actions. The sensor was positioned on the curved surface of the fingertip (Figure 2a). In order to uniformly distribute the force on the sensitive area of the sensor, the interface between the Gloreha sensor glove’s fingertips and the sensors was modified by inserting a thin 3D printed PLA plate ($1\times 10^{-3}$ m thickness and $2\times 10^{-2}$ m diameter), fixed to the sensor by means of double-sided tape (Figure 2b). Moreover, a PLA support was also printed in 3D and fixed to the connections of the flexible transmissions (see Figure 2c). The thin PLA plate also allows securing the sensors on the fingertips by means of an elastic band that can be fastened at the top of the custom-designed PLA support (Figure 2c).

The sensors attached to the developed PLA plate were calibrated using the Instron^®^ testing machine by applying forces in the range 0–8 N [29] and recording the corresponding output voltage by means of a custom made printed circuit board connected to the sensors.

### 2.2. Operation Mode I: Active-Assisted Training

The active-assisted training was developed with the aim of assisting the user in reaching a target grip force (i.e., the average force recorded on the three sensors exerted by the contralateral healthy hand of the patient). In this mode of operation, the actuators of the system were controlled based on the force information.

A preliminary calibration phase enabled us to acquire the maximum RoMs of fingers, measured by the flex sensors embedded in the glove, to guarantee that the flexion movements generated by the activation of the robot actuators in the active-assisted modality did not put the hand in a configuration which exceeded the subject’s functional RoM. In this calibration phase, the subject wears the glove with flexible transmissions mounted on the dorsum of the hand. Motors are moved via a custom-designed GUI to generate flexion of the fingers, and the finger configuration (i.e., the RoM) is measured through the embedded flex sensors. In this phase, the movement is free, and no object is grasped. The movement of the motor, which can be controlled by imposing incremental steps from 0 to 100 (i.e., the glove’s full range), is associated with the measured RoM before the subject starts experiencing discomfort. The measured RoM was used as a safety parameter in the active-assisted modality to prevent hand configurations that could be painful for a patient with limited RoM.

**Figure 3:**
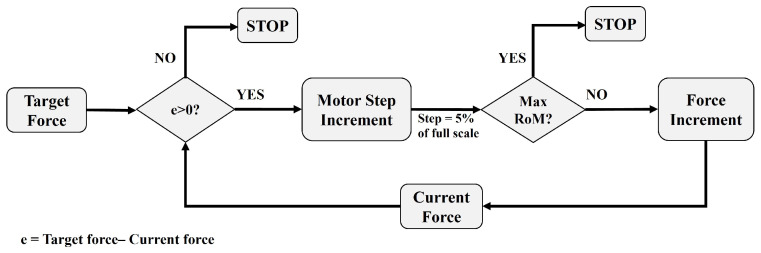
Block scheme of the strategy implemented for the active-assisted training. Every 3 s, the current force is compared with the target force. If the current force is lower than the target one, the position of the motor is updated by providing 5% of the full-scale position increment to generate additional grasping force. Moreover, the RoMs of the fingers are also evaluated every 3 s: if the RoM of one of the fingers exceeds the maximum RoM recorded in the calibration phase, the exercise ends.

The block scheme of the proposed strategy implemented for performing the active-assisted training is shown in Figure 3: At pre-set timesteps (i.e., every 3 s), the current force (i.e., the average force measured in real-time by the three sensors) is compared with the target force value set in the GUI. If the current force is lower than the target one, the position of the motor is updated with progressive incremental steps set to 5% of the motor’s maximum in order to provide additional grip force to the user and help to reach the target force value. Moreover, the RoMs of the fingers are also evaluated every 3 s: if the RoM of one of the fingers exceeds the maximum RoM recorded in the calibration phase, the exercise ends. This way, the system can assist the user only if in the predetermined time interval, he/she is not capable of reaching the target force. The motors provide a force contributing to the grasp in order to reeducate the user to exert target force levels. The choice of a 3 s time interval was guided by the fact that, in that interval, the Gloreha Sinfonia motors should activate, move, and stabilize, and this takes about 2 s. Then, the force applied to the fingertips has to be stabilized. Therefore, to ensure an accurate measurement of the force generated by the motor activation, it was not possible to go below 3 s.

A progress bar which fills according to the exerted force was also created, to be displayed on a desktop during the exercise. The active-assisted modality should be exploited to reeducate the user to reach target grip force levels in the first stages of the rehabilitation session.

### 2.3. Operation Mode II: Virtual Reality Game for Training and Evaluation

**Figure 4:**
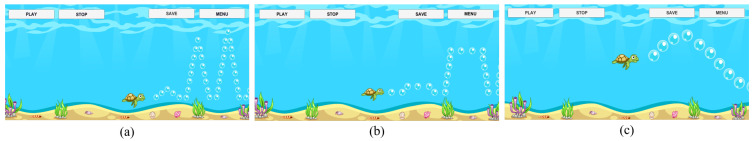
(**a**) “Ramp”, (**b**) “Square Wave”, and (**c**) “Sinusoid”.

A non-immersive VR game (Figure 4) was developed with the aim of assessing and training the user’s ability to (i) finely control the sub-maximal forces exerted in grasping tasks of real objects, (ii) balance and release grip, and (iii) accurately modulate the grip force [1]. This modality can be used in combination with the active-assisted training in a complete rehabilitative treatment or as a stand-alone modality to assess and train grip force control.

In this operation mode, the instrumented glove is passive, and motors are not exploited to generate additional force. According to the importance of biofeedback, attention was paid to provide intuitive, clear, and engaging visual feedback to the users. In the proposed VR game, the user controlled the avatar of the game which moved vertically according to the average force exerted by the three fingers during the grasping task. The purpose of the game was to track the proposed waveforms which moved on the screen from the right to the left, and to collect the maximum number of objects positioned onto them.

The VR game was developed with Unity, a game development platform which uses Microsoft Visual Studio as the editor and C# as the programming language.

Three different types of exercises were proposed in this work. They differ in the target waveform to be tracked by controlling the grip force and have different objectives:

1. The “Ramp” waveform was created with the aim of training and assessing the ability of the user to gradually increase and decrease grip force;
2. The “Square Wave” waveform was developed with the aim of training and assessing the ability of the user to exert discrete force levels and stabilize the force;
3. The “Sinusoid” waveform was created to improve the accuracy of the grip force control and evaluate the force modulation capability.

The “Ramp” (Figure 4a) waveform was composed of ascending and descending ramps to be tracked to reach 10 different force levels uniformly distributed between the minimum measured force (i.e., the force corresponding to a mere touch, without squeezing the object) and the maximum (or sub-maximal) grip force, previously set via the GUI. The 10 force levels followed each other in a random way.

The “Square Wave” (Figure 4b) was also composed of 10 discrete force levels to be reached and maintained, uniformly distributed between the maximum and minimum force and randomly administered.

The “Sinusoid” (Figure 4c) was characterized by a peak-to-peak amplitude which corresponded to the range between the maximum and minimum measured grip force, and a different frequency for each level of complexity.

In particular, for each waveform, three levels of complexity could be selected. The difficulty of the “Ramp” and “Square Wave” was meant to increase with the translation speed of the wave on the screen, whereas the difficulty of the “Sinusoid” was meant to increase with the frequency of the waveform.

The main menu screen of the VR game enabled us to set some parameters, such as the participant’s ID, the duration of the exercise, the level of difficulty, and the exercise to be administered. A timer on the upper part of the screen measured the duration of the exercise, and at the end of each trial, a summary of the performance was also shown. Data regarding the exerted force, the maximum force, and the minimum force were received via UDP communication from the custom-designed Microsoft Visual Studio application.

### 2.4. Experimental Validation

The enrolled volunteers provided written informed consent before participating in this study. The experimental protocol was approved by the local Ethical Committee (Comitato Etico Università Campus Bio-Medico di Roma, reference number: N. 41/17 OSS ComEt CBM) and complied with the Declaration of Helsinki.

#### 2.4.1. Operation Mode I: Active-Assisted Training

**Figure 5:**
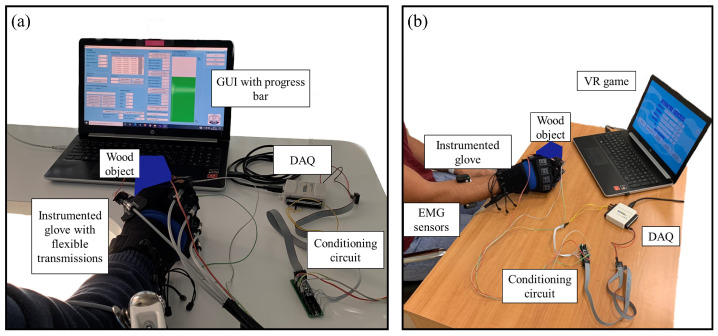
(**a**) Experimental setup for the active-assisted training; (**b**) experimental setup for the virtual reality game: electromyographic (EMG) sensors are positioned on the flexor digitorum superficialis and extensor digitorum communis muscles.

The experimental setup for the preliminary tests of the active-assisted training modality is shown in Figure 5a, where the personal computer (PC) showing the GUI, the Gloreha sensor glove with embedded force sensors and flexible transmissions, the conditioning electronics, and the DAQ USB—6008 device (National Instruments, Austin, TX USA) connected to the PC to power the conditioning circuit, are highlighted. The DAQ device has 8 analogue input pins (12 Bit, 10 kS/s), 2 analogue output pins, and 12 digital input/output pins. The output voltage coming from the conditioning circuit was acquired by means of the same DAQ device. A rate of 1000 Hz was set for the acquisition of the analogue output voltage.

As a proof-of-concept, the active-assisted modality was tested on three healthy male volunteers aged 30.3±1.1 years. The objective of the preliminary experimental tests was to evaluate the ability of the developed algorithm to control the Gloreha Sinfonia motors to generate additional grip force at the fingertips.

The participants sat on a chair in front of the monitor displaying the GUI with the visual feedback and wore the appropriately sized (i.e., M, XL, and XL, respectively, for the first, second, and third volunteers) Gloreha sensor glove with embedded force sensors and flexible transmissions on the dominant hand (i.e., right hand). Before executing the experimental trial, the maximum and minimum grip forces were computed by asking the volunteers to exert the maximum grip force on the supplied object three times. The maximum and minimum forces were calculated by averaging the forces exerted by the thumb, index finger, and middle finger in the three trials. Then, the volunteers were asked to grasp the object with their minimum grip force, without lifting it, and were instructed to remain passive during the whole trial. The object was a wood parallelepiped ($1.35\times 10^{-1}$ m $\times \;6.5\times 10^{-2}$ m $\times \;4\times 10^{-2}$ m), grasped with a precision grasp in the direction of its smaller dimension. The object’s dimensions were chosen to respect the optimal handle diameter to exert the maximum grip force [30]. The maximum force to reach during the experiment was set to 90% of the maximum recorded value. The maximum reachable positions of the motors were set to values which simulated limited RoMs (i.e., 60%). In fact, the maximum positions of the motors of this exoskeleton can be controlled using percentages of the maximum.

The trial started with the volunteer lightly gripping the supplied object. The participant was also instructed not to spontaneously increase the exerted grasp force during the trial, to take into account only the force increments provided by the actuation mechanism and measured by the force sensors on the fingertips. Every 3 s, the exerted force measured by the force sensors was compared to the target force. The positions of the motors were updated when the recorded force was lower than the target one. Motor position increments are expressed as percentages of the motor maximum position. In order to test the control strategy, increments of 5% every 3 s and a maximum reachable position of 60% were set. The progress bar shown in the GUI filled according to the force, providing visual feedback. The active-assisted training stopped when the maximum position of the motor was reached and/or the maximum target force was exerted either with or without the assistance provided by the motors. Once the exercise ended, the motors were moved to the home position and the volunteer could release the object. Six trials were executed for each volunteer, to evaluate whether the developed control algorithm could allow the motors to generate additional grip force during the exercise, with the participant being passive and not providing a spontaneous increase in grip force.

#### 2.4.2. Operation Mode II: Virtual Reality Game

The experimental setup for the VR game modality is shown in Figure 5b. It was similar to the setup used for the active-assisted training, but for this modality, the system was passive, and the instrumented Gloreha sensor glove was used without flexible transmissions because it only had to record grip forces exerted by the participant. Moreover, in this modality the PC was used to display the VR game. The analogue output voltage coming from the conditioning electronics was acquired at 1000 Hz using the previously described DAQ device. Acquired data were sent using the UDP protocol to Unity game development platform. Force and RoM data were sent and stored with a rate of 10 Hz to reduce the amount of data to be exchanged, still enabling us to finely control the VR game and store an informative amount of data for functional evaluation purposes. Moreover, as evidenced in [31], the briefest interval over which visual information could be integrated and used to correct errors in motor output is approximately 150 ms.

Superficial electromyographic (sEMG) data were acquired with the Delsys Trigno wireless sensors at 1000 Hz during the execution of the assigned tasks. sEMG were filtered with a sixth-order Butterworth bandpass filter with cutoff frequencies (30,450) Hz and then with a second-order Butterworth notch filter (50 Hz), and normalized with respect to the maximum voluntary contraction (MVC). sEMG signals from the flexor digitorum superficialis and extensor digitorum communis were acquired, as these represent the pair of agonist-antagonist muscles most involved in the assigned grasping task, as already confirmed in the literature [32]. sEMG signals were acquired with the aim of monitoring the level of muscle activation between the beginning and the end of the trial and of understanding whether the execution of the tasks did influence the muscular amplitude, due to muscular fatigue effects. In addition, the monitoring of muscular fatigue onset could also be used in future works to modify the percentage of maximum force to be reached by the patients during the execution of multiple exercises in a rehabilitation session, to further improve the proposed system.

In order to perform a preliminary validation of the proposed system for the training and assessment of grip force control and evaluate the developed VR games in terms of the possibility of extracting meaningful performance indicators to assess the user’s force control, 10 healthy volunteers (3 males and 7 females aged 26.1±1.8 years) were enrolled. All the volunteers wore a size M of the instrumented Gloreha sensor glove on the dominant hand (9 participants were right-handed and 1 left-handed). The glove was also regulated to comfortably fit the hand. The volunteers sat on a chair in front of a table on which the PC, showing the VR game, was placed. To perform the grasping task, the participants were asked to grasp, without lifting it, the same wood parallelepiped used in the active-assisted training validation.

Before running the VR game, the maximum and minimum grip forces were evaluated for each volunteer for the hand used to perform the exercise, with the same protocol followed for the active-assisted training. The maximum force to be reached in the VR game was set to 90% of the maximum recorded value to prevent the participant from being too tired when reaching and/or maintaining the highest force value during the trial. Similarly, the MVC was evaluated for each volunteer, by asking them to perform three maximal contractions of the two monitored muscles.

Once the maximum and minimum forces were set, the volunteers were asked to play the VR game. They were required to dynamically adapt the grip force exerted on the supplied object to track the three different waveforms moving on the screen and collect the greatest number of objects positioned on them, by vertically moving the avatar of the game according to the exerted force. In particular, to track the "Ramp" waveform, the volunteers had to gradually increase and decrease the exerted force and reach the peaks of the ramp. For the "Square Wave" waveform, the participants had to reach discrete force levels and stabilize the force, whereas the aim of the "Sinusoid" was to track the waveform by gradually modulating the force.

Before starting the trial, the volunteers performed an initial phase of familiarization in which they executed a one-minute repetition of each type of exercise with the lowest level of difficulty. After the familiarization, they performed 4 repetitions of each of the three levels of complexity for the proposed waveforms. Each repetition lasted 1 min. The different waveforms and levels of complexity were administered randomly to reduce habituation. Between each repetition, the participants rested a few seconds according to their needs. The entire trial lasted about 1 hour per volunteer.

At the end of the trial, the NASA TASK LOAD INDEX (TLX) questionnaire was administered to each volunteer, to evaluate the usability of the proposed VR games through the computation of Overall Workload (OW) of the task [33]. The participants assigned a weighted rating in the range 0–100 with 5-point steps to each of the six subscales (Mental Demand, Physical Demand, Temporal Demand, Performance, Effort, Frustration). The ratings were then combined to obtain the OW, which is represented by a score from 0 (low OW) to 100 (high OW) [34]. Moreover, in order to collect the subjective impressions of the participants with respect to the performed experiment, an additional questionnaire was administered to each volunteer at the end of the trial. Participants had to assign a rating from 1 to 5 to each of the following questions:

(*Q*1) How much did you enjoy your experience with the VR game? (1 not at all–5 very much);
(*Q*2) How would you rate the level of intuitiveness of the VR game, i.e., the mechanism which transduces forces to the avatar movement? (1 not intuitive–5 very intuitive);
(*Q*3) How much did the VR game involve you? (1 I did not feel involved at all–5 I was really involved and the VR game maintained high my attention during the experiment);
(*Q*4) How would you rate the level of comfort of the instrumented glove?—i.e., was the device comfortable when worn? (1 not comfortable–5 comfortable);
(*Q*5) How natural was the mechanism which controlled movement through the environment? (1 extremely artificial–5 completely natural);
(*Q*6) How responsive was the environment to actions that you performed? (1 not responsive–5 completely responsive).

In addition to the validation on healthy volunteers, the VR game was tested on a pediatric patient (i.e., a 10-years old girl) with right-hand hemiparesis following ischemic stroke [35]. The grip force control capability of the patient was assessed two times in three months, before and after robot-mediated therapy with Gloreha Sinfonia. In each evaluation session, the maximum and minimum grip forces were measured according to the previously explained protocol. Then, the patient was asked to perform the tracking tasks of the three proposed waveforms. The maximum force to reach was set to 90% of the maximum recorded value. In particular, she performed multiple one-minute repetitions of the different exercises (i.e., 5 repetitions of "Ramp" and 3 repetitions of "Square Wave" and "Sinusoid" in the first session, and 3 repetitions of "Ramp" and 2 repetitions of "Square Wave" and "Sinusoid" in the second session).

#### 2.4.3. Performance Indices

For the active-assisted modality, the force variation generated by the activation of the motors of the thumb, index finger, and middle finger, and measured by the three sensors positioned at the fingertips, was saved for each trial together with the incremental steps of each motor’s position. The average force exerted by the motors on the three sensors was then computed, and the mean force in each 3 s interval corresponding to a given motor activation step was calculated, so as to obtain 13 force values (i.e., one for each motor step from 0% to 60%). The correlation between the average force values and the motor steps was considered as a performance index (PI) for this modality, to understand whether the actuators of the device were able to generate additional grasping force.

The following PI were evaluated to assess the volunteers’ performance in the VR game:

1. Performance (P) (%) of each volunteer in every repetition for the three waveforms with three levels of complexity (C), computed as the number of collected objects with respect to the total number of objects.
2. Peak Performance (PP) (%) of each participant in every repetition at each level of complexity (C) of the three waveforms. It considers the upper peaks of the waveforms reached for the "Ramp" and "Sinusoid" waveforms, and the reached and held force levels for the "Square Wave" (i.e., the constant discrete force levels). It is computed as the number of collected objects over the total number of objects on the peaks and discrete force levels.
3. Normalized tracking error (TE) (-), computed as the average force error between the target force pattern to be tracked and the force exerted by the volunteer during the trial, and extracted for each repetition of the three levels of the waveforms for all the participants.
4. Normalized root mean-square error (RMSE) (-) between the target waveform and the volunteer’s force pattern, calculated for each participant in every one-minute repetition of the task.
5. OW of the task for each volunteer.

In order to allow comparison among the results obtained for different volunteers, the TE and the RMSE for each participant were normalized by the difference between the maximum and minimum force acquired for each volunteer, according to [11], to obtain a number between 0 and 1. Therefore, these indicators are dimensionless.

Finally, to compare the amplitudes of the muscular contraction between the beginning and the end of the trial and investigate the possible onset of muscular fatigue, the root mean-square level (rms) of the EMG signal of the two monitored muscles was computed for each volunteer at the beginning and at the end of the trial, in the same conditions (i.e., same limb configuration, same force level to be exerted, and same type of task) [36]. In fact, for the same experimental conditions, an increase in the amplitude of the EMG signal means that a greater number of muscular fibers is recruited for the desired contraction. This could be related to the onset of muscular fatigue [37]. This type of analysis was conducted to understand whether the onset of muscular fatigue affected the performance of the participant.

#### 2.4.4. Statistical Analysis

The data collected on healthy volunteers were used to carry out a statistical analysis, in order to investigate the existence of correlations between the indices defined in Section 2.4.3 and to verify if the implemented exercises and indicators allowed assessing the improvements of the participant in controlling and modulating the grasping force. Table 1 presents the pairs of variables considered for the correlation and statistical analysis.

Specifically, the following expressions were used to represent the averages of the P, PP, and TE considered to perform the analysis: average total denotes the mean of the considered PI over the entire trial consisting of 4 repetitions of each waveform for each of the 3 levels of complexity, computed for each volunteer, whereas average communal denotes the mean of the considered PI for all the 10 healthy volunteers for each one-minute repetition, computed for each level of complexity of each waveform.

The correlations between OW and average total P, OW and average total TE, maximum grip force and average total P, maximum grip force and average total PP, maximum grip force and average total TE (for the VR game modality), and force increments and motors positions (for the active-assisted modality) were investigated through the computation of the Spearman’s rank correlation coefficient (ρ). This test is suitable for non normally distributed data (as in this case) and assesses how well an arbitrary monotonic function can describe the relationship between two variables (i.e., the pairs of arrays of the PIs obtained by the 10 volunteers). The correlation is considered “very weak” if 0.0≤|ρ|≤0.19, “weak” if 0.20≤|ρ|≤0.39, “moderate” if 0.40≤|ρ|≤0.59, “strong” if 0.60≤|ρ|≤0.79, and "very strong" if 0.80≤|ρ|≤1.0 [38]. The Spearman’s rho is equivalent to Pearson’s linear correlation coefficient applied to the rankings of the two variables arrays. The test also returns the *p*-value (*p*): if p<0.05 the correlation is significantly different from zero.

Given that the level of complexity C is a 3-level categorical variable, the relationship between C and average communal P, and that between C and average communal TE, were investigated through the Kruskal–Wallis test, a nonparametric version of classical one-way ANOVA, which is suitable for the data collected in this experiment, as they exhibit a non normal distribution. This test evaluates the medians of the groups of data to determine whether the samples (i.e., the scores of the considered PI for each complexity level) come from the same population—that is, whether the factor (i.e., the level of complexity C) has a significant effect on the computed PI. The significance level was set to 0.05. This analysis was performed for each proposed waveform in order to understand whether different levels of complexity entailed different performance.

## 3. Results

In this section, results about the two tested operation modes are provided. The data post-processing and analysis were performed in MATLAB (R2021b).

### 3.1. Operation Mode I: Active-Assisted Training

The average force increments (i.e., difference between maximum and minimum mean force on the six trials) generated by the motors at the fingertips of the thumb, index finger, and middle finger, and the total force increments (i.e., the sum of the three forces), together with the range of the correlation coefficients obtained on the six trials, which express the strength of the monotonic relationships between the motor step and the average force increment for the three fingers, are shown in Table 2 for the three volunteers. For all the trials, p<0.05 were obtained.

**Figure 6:**
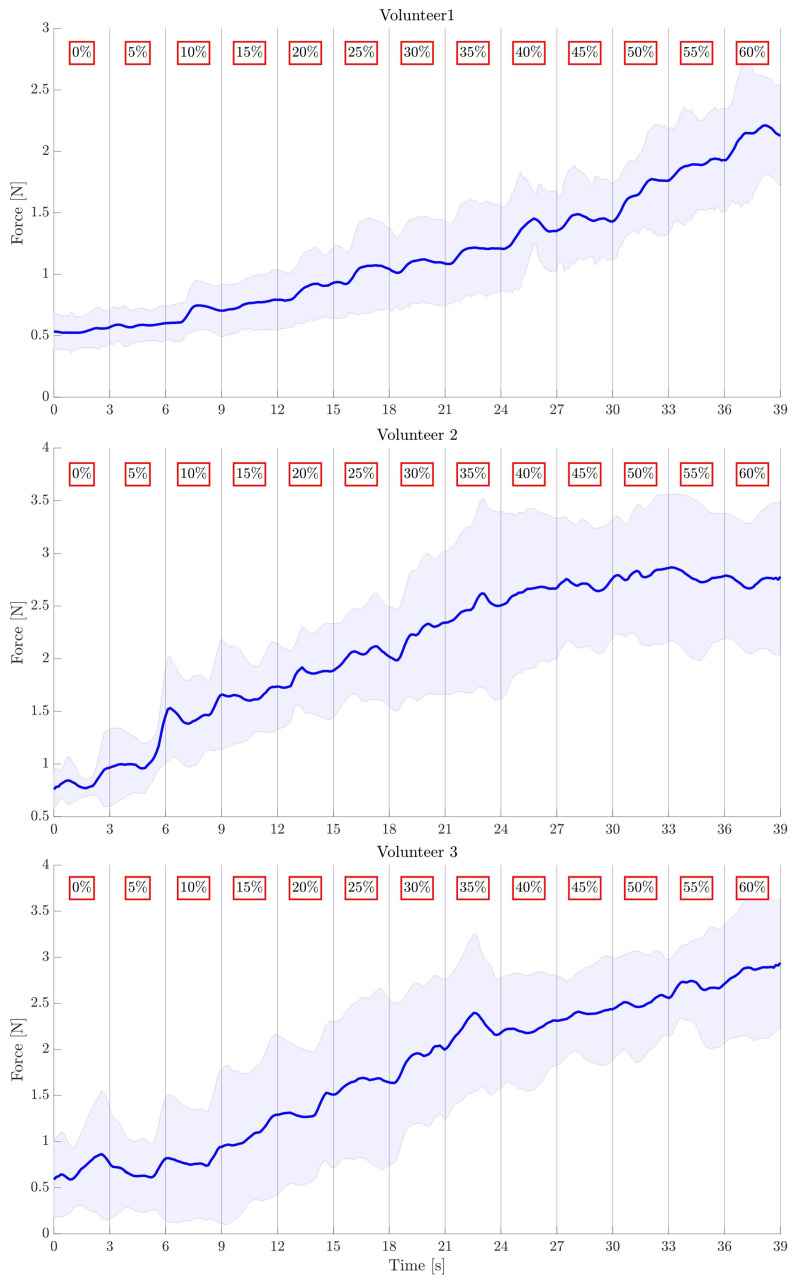
Average force increment (blue line) ± standard deviation (shaded area) for the 6 trials for each volunteer. The percentages in the red squares indicate the motors’ steps with respect to the maximum value.

Figure 6 shows the increasing trend of the average force exerted on the three fingers by the motors, together with the motor step, for each volunteer. In particular, the blue line represents the mean of the average force in the six trials, and the shaded area represents the standard deviation.

### 3.2. Operation Mode II: Virtual Reality Game

#### 3.2.1. Experimental Tests on Healthy Volunteers

**Figure 7:**
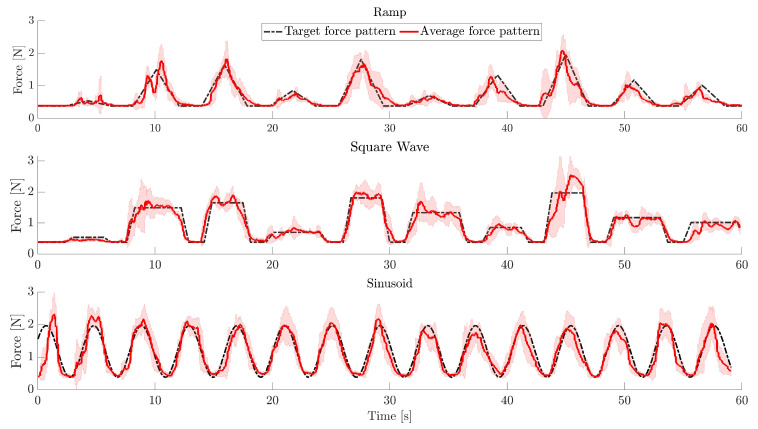
Average force pattern (red line) ± standard deviation (shaded area) and target force pattern (black dotted line) for the “Ramp”, “Square Wave”, and “Sinusoid”, level 1, for one representative volunteer who obtained average performances among the enrolled volunteers. The average force and standard deviation were computed for the 4 repetitions of level 1 for each exercise.

As a graphical example of the executed task, Figure 7 shows the average force pattern superimposed on the target force pattern for one representative healthy volunteer.

The average total P, average total PP, average total TE, and OW scores for the 10 volunteers, with the maximum grip force values, are given in Table 3. Results about the RMSE obtained by the volunteers are similar to the average total TE results and are not reported for brevity.

The Spearman’s rank correlation coefficients and *p*-values obtained from the statistical analysis are given in Table 4.

**Figure 8:**
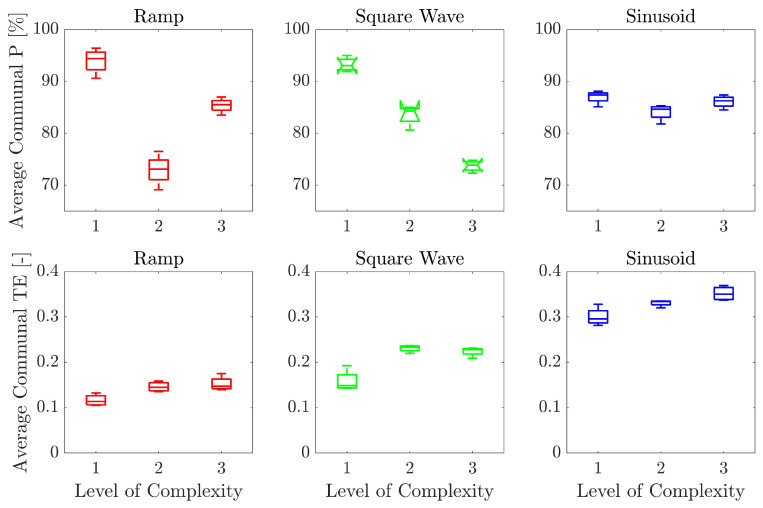
Boxplot of the average communal P and average communal TE for the three waveforms at each level of complexity. The central line is the median of the scores of the 10 volunteers in each one-minute repetition for every level of complexity, the edges of the box are the 25th and 75th percentiles, and the whiskers extend to the most extreme data points not considered outliers.

Figure 8 shows the average communal P and average communal TE scores obtained by the 10 volunteers in each one-minute repetition at each of the three levels of complexity, for the three proposed waveforms. For the C/average communal P pair, the Kruskal–Wallis test returned *p*-values of 0.01, 0.01, and 0.06 for the “Ramp”, “Square Wave”, and “Sinusoid”, respectively, whereas for the C/average communal TE pair, the test returned *p*-values of 0.02, 0.02, and 0.01 for the “Ramp”, “Square Wave”, and “Sinusoid”, respectively.

**Figure 9:**
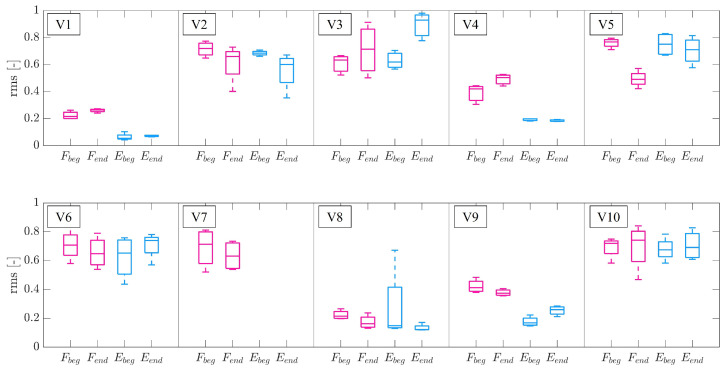
Root mean-square level (rms) of EMG signals for each volunteer (V). $F_{beg}$ and $F_{end}$ are the indices computed on flexor digitorum superficialis (in magenta), and $E_{beg}$ and $E_{end}$ were computed on extensor digitorum communis (in light blue), at the beginning and the end of the trial, respectively. The middle line is the median of four trials in the same experimental conditions, the edges of the box are the 25th and 75th percentiles, and the whiskers extend to the most extreme data points not considered outliers.

The analysis conducted on the rms of the EMG signals did not report statistically significant differences in the amplitude of the signal for all the participants, for the same experimental conditions. Figure 9 shows the boxplot of the computed indices for the 10 volunteers.

Finally, results about the mean and standard deviations of the subjective questionnaire administered to the 10 healthy volunteers are shown in Table 5.

#### 3.2.2. Hemiparetic Patient

To assess the grip force control and modulation capability of the patient, the normalized RMSE, the P, and the PP were calculated for each exercise. In particular, the RMSE was normalized with respect to the maximum force applied by the patient in each session.

To evaluate the improvement of the patient in grip force control, results were compared between the two assessment sessions (i.e., before and after the robot-mediated therapy) by applying the Wilcoxon signed-rank test, with a 5% significance level.

**Figure 10:**
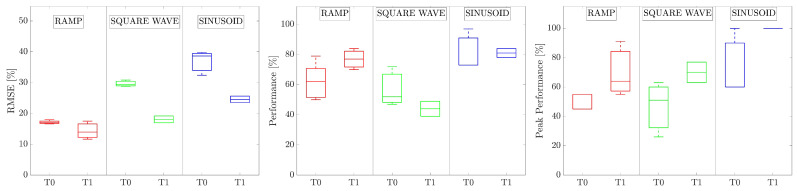
Normalized RMSE expressed as a percentage of the maximum force, P, and PP of the patient for each waveform. The middle line is the median computed for the executed repetitions of each waveform, the edges of the box are the 25th and 75th percentiles, and the whiskers extend to the most extreme data points not considered outliers.

Figure 10 shows the normalized RMSE, the P, and the PP obtained for each of the three waveforms, before and after the therapy (T0 and T1, respectively). The three waveforms are highlighted with different colors.

The *p*-values obtained from the Wilcoxon signed-rank test were p=0.016, p=0.922, and p=0.031 for RMSE, P, and PP, respectively.

## 4. Discussion

The results presented in Section 3.1 demonstrated that the force increments produced by the motors and the motor steps in each trial are “very strongly” positively correlated in a statistically significant manner. Therefore, the developed control strategy for the active-assisted modality could drive the motors of the Gloreha Sinfonia system to generate additional grasping force when they are moved of fixed steps, to assist the participant in reaching a target force level. The absolute force increments from the resting condition in the first, second, and third finger were in the order of 2 N, and the total force generated by the motors ranged from 5 to 7 N for the three volunteers. Given that the masses of the most common objects used in ADLs are below 1 kg, which translates to ∼10 N, the generation of a force in the order of about 2 N per finger is enough for grasping most of the objects used in ADLs [39,40].

The maximum grip forces exerted by the volunteers in the VR game were below 5 N (see Table 3). These forces were not particularly demanding to be reached, and matched the range of forces typically used in ADLs (i.e., around 10 N [39,40]). In addition, the maximum duration for which the maximum grip force should be maintained among all the proposed exercises is less than 3 s, and this occurred in the “Square Wave” exercise, level 1.

As highlighted in Table 4, the “moderate” negative correlation between average total P and OW showed that the performance decreased when the perceived OW of the task was higher. The “weak” positive correlation between average total TE and OW supported the previous result. The "moderate" positive correlation between maximum grip force and average total P could imply that when the maximum grip force recorded for each volunteer was higher, the performance also increased. This suggests that regulating the force within a wider range could be easier for the participants, resulting in better performance. Similarly, the “moderate” negative correlation between maximum grip force and normalized average total TE supported the previous assumption. In fact, lower tracking errors were recorded if the maximum grip force was higher, and this suggested that scaling the force in a wider range could be simpler, also considering the fact that only healthy volunteers, who did not report any problem in exerting high grip forces, were tested. Moreover, the "weak" positive correlation between the maximum grip force and average total PP suggested that the capability of reaching and holding discrete force levels did increase when the measured maximum grip force was higher, accordingly to the easiness of regulating the force in a wider range.

The relationship between the average communal P and average communal TE scores for the different levels of complexity is presented in Figure 8. The average communal P scores tended to decrease as the level of complexity increased. This is evident for the “Square Wave” exercise, whereas for “Ramp”, the performance decreased between level 1 and 2, and increased again between level 2 and 3. For the “Sinusoid”, there is no visible decreasing trend of performance. However, these differences for the "Sinusoid" were not statistically significant. As expected, in the developed exercises, the increasing complexity of the levels caused diminution of the performance for the “Square Wave” exercise, and partially for the “Ramp” exercise. It is worth highlighting that P, as a PI, considers only the number of collected objects with respect to the total number of objects in the scene. This entails that objects could be collected even though the target force pattern is not followed, and the avatar of the game is moved randomly. Therefore, besides the P, more precise PI should be always analyzed to provide exact information about the difference between the target and executed force pattern as a continuous signal over time. These indicators are the TE and RMSE. In fact, the same type of analysis performed on the average communal TE showed a general increasing trend of the TE with the level of complexity, for the three exercises. The differences in the scores between the levels of complexity were statistically significant for all the exercises. This result strengthens the hypothesis that an increasing level of complexity entails higher tracking errors for all the volunteers. Therefore, the design choice of increasing the complexity of the task based on translation speed (for the “Ramp” and “Square Wave”) and frequency (for the “Sinusoid”) was a correct way of defining exercises and difficulty levels.

The high standard deviations obtained for average total TE, reported in Table 3, could be explained by the fact that the difficulty is different among the levels and increases with an increasing level of complexity; this entails an increase in TE, as also confirmed by the results obtained for average communal P.

The analysis conducted on the EMG amplitude for each participant implied that there is no evidence that the onset of muscular fatigue altered the performance of the volunteers. This aspect was also confirmed by the conducted statistical analysis.

In general, all the volunteers assigned high scores in the subjective questionnaire. All the participants rated with high scores the level of intuitiveness of the VR game and the responsiveness of the environment. The first question received a score of 3 from volunteer 3, who also reported that one hour of the experiment was particularly demanding. However, this trial lasted one hour to test all the proposed exercises, but patients are not required to execute all of them. They only perform the exercises with the waveform, level of complexity, and percentage of maximum grip force to be reached that are most appropriate for their conditions. Nevertheless, patients could perform the complete trial by including pauses between the exercises, and the monitoring of muscular fatigue onset could be used to modify the percentage of maximum force to be reached to prevent overexertion. The level of involvement was rated with an average score of 4.1/5 for the volunteers; therefore, the participants maintained high their attention throughout the trial. Q4, related to the level of comfort of the glove, received the lowest cumulative score (3.8/5 on average) from the participants. Anyhow, the integration of the force sensing resistors on the device was stable, and the participants only reported a bit of discomfort because they had to wear the device for about one hour.

The results obtained for the hemiparetic patient allowed quantifying the improvements in grip force control and modulation capability after robot-mediated therapy. The improvements were in agreement with the functional scales (i.e., Fugl–Meyer Assessment—Upper Extremity) administered to the patients [35]. In particular, the RMSE significantly decreased (p=0.016) and the PP significantly increased (p=0.031) between the two evaluation sessions. The RMSE allowed quantifying improvements in the modulation of grip force control, whereas the PP allowed quantifying the amelioration of the capability of reaching and holding discrete force levels and the force stabilization. On the other hand, results on the P were not statistically significant. In fact, as previously highlighted, P considers only the number of collected objects, whereas the RMSE provides more precise information about the tracking error executed during the whole trial. These results suggest that the TE and the RMSE are the most reliable indicators to be considered for quantifying the users’ performance. In addition, the young patient reported enjoying the VR game and feeling engaged during the assessment session.

## 5. Conclusions

This paper described the development and validation of a system for the assessment and recovery of grip force control. A commercial exoskeleton for hand rehabilitation was instrumented to sense the grip force exerted by the fingers when grasping real objects. VR games for the training and assessment of grip force control were developed and tested on healthy volunteers and a hemiparetic patient. A force-based, active-assisted modality was also proposed and preliminarily validated on healthy subjects.

The preliminary tests of the active-assisted modality showed that the motors could generate force increments in an order of magnitude which allows grasping and manipulating objects used in ADLs. This preliminary validation proves the feasibility of implementing an active-assisted training in force with Gloreha Sinfonia and paves the way for a further refinement of this modality. Additional tests will be conducted on healthy subjects and patients in future works.

The VR game proved to be intuitive and engaged the volunteers during the training session. The experimental validation allowed verifying whether the proposed PIs were able to quantitatively assess the performance of the user during the treatment. The choice of increasing the level of complexity of the VR games by increasing the speed and frequency of the waveforms proved to be successful. Therefore, different complexities could be selected when administering the exercises to patients, according to their conditions.

Future works will be devoted to refining the active-assisted training modality. To evaluate the clinical utility of the proposed grip force training system, additional tests on patients will be conducted in the future. In addition, the aspects related to force synergies theory and motor control strategies in grasping tasks will be further investigated.

## Figures and Tables

**Table 1:** Correlation between pairs of variables.

Pair	Reason for the Investigation
OW and average total P	To understand whether who scored lower P perceived a higher OW
OW and average total TE	To understand whether who perceived a higher OW also made greater TE
Maximum grip force and average total PP	To investigate whether higher forces to reach could decrease the capability of reaching peak force values and hold force
Maximum grip force and average total P	To understand whether higher forces were correlated to an increase or a decrease in P
Maximum grip force and average total TE	To investigate whether higher forces were correlated to higher or lower TE made by the volunteers
C and average communal P	To understand whether, overall, the increasing level of complexity could lead to a diminution of P
C average communal TE	To understand whether, overall, the increasing level of complexity could lead to an increase in the TE
Force increment and motors position	To assess whether the system motors could generate grip force increments starting from resting conditions

**Table 2:** Force increments generated by the motors and correlation coefficients for each volunteer.

Volunteer	Thumb Force [N]	Index Force [N]	Middle Force [N]	Total Force [N]	ρ
1	1.88	1.05	2.14	5.1	0.96–0.99
2	2.35	1.60	2.80	6.75	0.94–0.99
3	3.38	1.94	1.99	7.31	0.85–0.99

**Table 3:** ATP (%), ATPP (%), ATTE (-), OW (-), and maximum grip force F (N) for each volunteer (V).

	ATP	ATPP	ATTE	OW	F
V1	91.0 ± 6.9	93.7 ± 9.5	0.16 ± 0.17	34	3.93
V2	82.3 ± 9.3	71.8 ± 19.0	0.22 ± 0.24	50	3.34
V3	83.9 ± 9.7	78.3 ± 19.2	0.16 ± 0.18	43.3	2.91
V4	89.0 ± 8.2	84.9 ± 12.0	0.13 ± 0.14	77.3	4.45
V5	82.3 ± 8.8	73.2 ± 13.9	0.67 ± 0.71	66	1.45
V6	85.1 ± 8.5	76.1 ± 23.7	0.16 ± 0.17	49.7	3.49
V7	80.3 ± 10.0	69.6 ± 23.3	0.21 ± 0.22	58.7	3.15
V8	93.5 ± 5.9	95.3 ± 7.6	0.14 ± 0.16	43	4.72
V9	86.1 ± 8.7	85.2 ± 13.8	0.15 ± 0.17	56	1.97
V10	70.9 ± 13.7	48.2 ± 29.0	0.24 ± 0.26	61.7	4.13

**Table 4:** Spearman’s rank correlation coefficients and *p*-values.

Pair	ρ	*p*
OW and average total P	−0.50	0.14
OW and average total TE	0.24	0.51
Maximum grip force and average total PP	0.27	0.45
Maximum grip force and average total P	0.42	0.23
Maximum grip force and average total TE	−0.49	0.15

**Table 5:** Mean and standard deviation of the subjective questionnaire given to the 10 volunteers.

Question	Mean ± Standard Deviation
Q1	4.20 ± 0.63
Q2	5.00 ± 0.00
Q3	4.10 ± 0.74
Q4	3.80 ± 0.79
Q5	4.80 ± 0.42
Q6	4.90 ± 0.31

## Data Availability

Not applicable.
